# Knowledge, Attitude, Practice of Dengue Fever and Its Related Factors among International Students at Tehran University of Medical Sciences

**Published:** 2018-11

**Authors:** Kourosh HOLAKOUIE-NAIENI, Owais RAZA, Shahrzad NEMATOLLAHI, Leonard EMMANUEL MENSAH, Javed MEMON, Shema MHAJABIN

**Affiliations:** Dept. of Epidemiology and Biostatistics, School of Public Health, Tehran University of Medical Sciences, Tehran, Iran

## Dear Editor-in-Chief

Dengue virus (DENV) infection has globally become a major public health concern, since the incidence of Dengue Fever (DF) has increased more than 30-fold over the last five decades and the disease is now endemic in 128 countries. Overall, 390 million DENV infections are estimated to occur per year, which is more than three times of previous estimates by the WHO (1). Iran, with approximately 80 million population, is the second-largest nation in the Middle-East, where DF is considered an emerging disease (2). Tehran University of Medical Sciences (TUMS), the largest and oldest university of medical sciences in Iran, is now hosting a total of 685 international students, mostly citizens of dengue-endemic countries such as middle-eastern and northern-African countries.

Hence, the investigation of Knowledge, Attitude and Practice (KAP) of international students affiliated to TUMS regarding prevention of dengue fever seems necessary. Therefore, the study aimed to determine the KAP and its related factors about prevention of Dengue fever among international students at TUMS.

This cross-sectional study was carried out on a convenient sample of 30 international students at the School of Public Health during spring 2017. A self-structured questionnaire was developed and its reliability and validity were tested using Cronbach’s alpha. The questionnaire included information on socio-demographics, Knowledge, Attitude, and Practice (KAP). For KAP part, each correct answer was given a score of one, and using visual binning, knowledge and attitude was divided into three categories (poor, medium, and average), and practice into two categories (low and high). Considering the confounders such as age, sex, education, and place of residence; multivariate analyses were performed to adjust the associations between KAP and each socio-demographics. Informed consent was sought verbally.

SPSS version 22 (Chicago, IL, USA) and Microsoft Excel (2013) was used for data analysis.

Table 1 gives a brief description of the study variables. The mean age was 32.34 (5.61). Most of the participants (90%) were urban dwellers, 43.3% had a low expenditure which is less than $100. Nineteen of the participants (65.5%) came from the African endemic region. Out of 30 participants, the mean scores of knowledge, attitude, and practice were 17.0, 2.10, and 4.83 respectively.

**Table 1:** Descriptive statistics of knowledge, attitude, and practice score with socio-demographics of the study participants

***Dependent Variables***		***Mean***	***Standard Deviation***
Knowledge Score		17.0	5.9
Attitude Score		2.10	0.81
Practice Score		4.83	1.09
Age (Independent)		32.34	5.61
**Independent Variables**		**Frequency**	**Percentages**
Gender	Male	22	73.3
	Female	8	26.7
Marital Status	Single	12	40
	Married	18	60
Previous Level of Education	Undergraduate	10	33.3
	Post-graduate	20	66.7
Place of Residence	Urban	27	90
	Rural	3	10
Monthly Expenditure	Low (< $100)	13	43.3
	Medium ($100–$300)	12	40.0
	High (>$300)	3	10.0
Occupation	Working	15	50
	Not-working	14	46.7
Regions (Before coming to Iran)	Africa	19	65.5
	Asia	3	10.3
	Middle-East	7	24.2

The distribution of knowledge and attitude scores was significantly related to different demographic characteristics. Specifically, the proportion of good knowledge was lowest in undergrad students, and highest in master students (11.1% vs. 36.8%, *P*=0.08). The proportion of good attitude was highest in urban dwellers and lowest in rural ones (37% vs. 0%, *P*=0.036). On the other hand, the distribution of practice did not show any pattern according to demographics (Table 2).

**Table 2:** Association between Knowledge, Attitude and Practice with selected demographics in the study participants

***Variables***		***Knowledge (%)***				***Attitude (%)***				***Practice (%)***		
		***Poor***	***Ave.***	***Good***	***P***	***Poor***	***Ave.***	***Good***	***P***	***Poor***	***Good***	***P***
Gender	Male	36.4	31.8	31.8	0.39	27.3	36.4	36.4	0.51	77.3	22.7	1.0
	Female	62.5	25	12.5		50	25	25		75	25	
Place of Residence	Urban	44.1	25.9	29.6	0.29	25.9	37	37	0.036	74.1	25.9	0.44
	Rural	33.3	66.7	0		100	0	0		100	0	
Previous Level of Education	Undergrad	33.3	45.6	11.1	0.08	22.2	33.3	44.4	0.59	88.9	11.1	0.25
	Masters	47.4	15.8	36.8		36.8	36.8	26.3		68.4	31.6	
Monthly Expenditure per Month	Low (< $100)	46.2	23.1	30.8	0.56	38.5	23.1	38.5	0.33	76.9	23.1	0.48
	Medium ($100–$300)	25	41.7	33.3		16.7	50	33.3		66.7	33.3	
	High (>$300)	39.3	32.1	28.6		66.7	33.3	0		100	0	

Findings of the present study are in accordance with previous studies reporting that previous level of education (1,3) and place of residence (1) are important factors of KAP regarding DF.

Despite the small sample size and its inherent methodological flaws, the present small-scale study at the School of Public Health showed that international students did not have enough KAP regarding dengue fever. Therefore, proper educational programs to increase the level of awareness, and culturally adjusted programs to enhance the level of attitude and practice are warranted.
